# The Immunomodulatory Role of Galectin-1 in the Tumour Microenvironment and Strategies for Therapeutic Applications

**DOI:** 10.3390/cancers17111888

**Published:** 2025-06-05

**Authors:** Alice Griffiths, Palita Udomjarumanee, Andrei-Stefan Georgescu, Muruj Barri, Dmitry A. Zinovkin, Md Zahidul I. Pranjol

**Affiliations:** 1 Department of Biochemistry, School of Life Sciences, University of Sussex, Brighton BN1 9QG, UK; 2 School of Biological Sciences, Faculty of Environmental and Life Sciences, University of Southampton, Southampton SO17 1BJ, UK; 3 Department of Pathology, Gomel State Medical University, 246000 Gomel, Belarus

**Keywords:** galectin-1, tumour microenvironment, immunosuppression, T cell suppression, tumour associated macrophages, therapeutics

## Abstract

With the morbidity of cancer currently on a perpetual rise, there is a critical need for new treatment options. Current therapeutic options, such as chemotherapy and radiotherapy, are frequently employed; however, the high rate of recurrence underscores the incomplete understanding of tumour growth, progression, and the intricacies of their microenvironments. In this study, we review the roles that galectin-1 (Gal1) plays in suppressing immune surveillance in the tumour microenvironment. Studies have shown that Gal1 changes the immune system parameters: suppressing T cell function, sensitising resting T lymphocytes to Fas/FasL, decreasing cell proliferation, reducing adhesion to extracellular matrix, inhibiting Th1 cytokines, increasing M2 phenotype macrophages, and promoting angiogenesis. Gal1 has garnered increasing attention as a potential therapeutic target due to its involvement in tumour progression and immune evasion. Given the limitations and toxic side effects associated with current treatment options, alternative strategies targeting Gal1 have been explored for their therapeutic potential. Approaches such as OTX008, anti-Gal1 monoclonal antibodies, and Gal1-targeted vaccines have demonstrated the ability to downregulate tumour progression by inhibiting Gal1 activity. These findings highlight the therapeutic promise of Gal1 not only as a novel target for cancer therapy but also as a potential prognostic biomarker, offering opportunities for the development of more effective and less toxic treatment strategies.

## Simple Summary

Cancer cases are rising, and current treatments like chemotherapy and radiotherapy often fail to prevent recurrence. This may be due to gaps in our understanding of how tumours grow and interact with their surroundings. This review focuses on galectin-1 (Gal1), a protein that helps tumours evade the immune system by weakening T cell responses, altering cytokine production, and promoting tumour-supporting immune cells and blood vessels. Because of its key role in tumour progression and immune evasion, Gal1 is being explored as a new treatment target. Promising strategies, including drugs, antibodies, and vaccines that block Gal1, may offer more effective and less toxic alternatives to current therapies. Gal1 also shows potential as a biomarker for cancer prognosis.

## 1. Introduction

Cancer affects 391,000 individuals in the UK alone every year and is the leading cause of death worldwide [1]. With a prevalence of 3 million people currently living with cancer in the UK and a survival rate of just 50%, the demand for more effective therapeutic intervention has never been greater [1]. Despite new treatments resulting in increased survival rates, a significant gap remains between treatment and curative measures [1]. Immune surveillance represents a critical component of the host’s defence against neoplastic transformation and progression. Among the myriad of factors influencing tumour dynamics, the dysregulation of immune responses is strongly associated with poor clinical outcomes.

The immune system comprises both innate and adaptive branches, which function synergistically to maintain immune cell homeostasis and mount effective anti-tumour responses [2]. However, tumours have evolved mechanisms to evade this immune surveillance, often through the exploitation of immunosuppressive pathways. Among the molecules involved in this process are galectins, a conserved family of β-galactoside-binding lectins characterised by their specific affinity for carbohydrate moieties and the presence of a conserved carbohydrate recognition domain (CRD) composed of homologous amino acid sequences [3]. Galectins have emerged as key modulators of tumour–immune interactions [4], with Galectin-1 (Gal1) being the most well-studied member.

Gal1 was the first identified member of the galectin family, originally discovered in 1975 from the electric organs of the electric eel Electrophorus electricus [5]. Gal1 is a small ubiquitously expressed lectin found in both physiological and pathological contexts, where it participates in diverse biological processes, including cell proliferation, migration, immune modulation, and angiogenesis. Notably, Gal1 has been implicated in promoting tumour progression, immune escape, and metastatic dissemination [6]. Gal1 has been shown to subvert immune responses by engaging inhibitory receptors on immune cells and modulating their activity, thereby promoting immune evasion [5]. This immunosuppressive capacity contributes to tumour proliferation, local invasion, and ultimately metastatic dissemination, reinforcing the role of Gal1 in promoting tumour progression through the circumvention of immune-mediated control. This review will focus on elucidating the immunomodulatory functions of Gal1 across various stages of cancer progression and will critically assess its potential as a target for therapeutic intervention.

## 2. Pathological Function of Gal1

Gal1 has been shown to play key roles in cancer progression and angiogenesis [5,7]. Cells within the tumour microenvironment (TME), such as tumour-infiltrated immune cells, endothelial cells (ECs), and tumour cells, are known to secrete Gal1, which functions as a pro-tumorigenic factor [2,5,7]. The presence of Gal1 has, therefore, been suggested as a biomarker for malignant tumour progression [8]. The upregulation of Gal1 has been observed in several cancer types, including lung, breast, ovarian, and prostate cancers, as shown in Table 1 [5]. Thus, with Gal1 evidently playing a key pathological role in tumours with its upregulation in such a vast mass of cancers, the underlying biological processes must be studied in aid of improving prognosis for patients.

Gal1 has emerged as a pivotal regulator of immune suppression within the TME. While Gal1 is known to contribute to the resolution of inflammation through its immunosuppressive functions under physiological conditions, malignant cells and associated stromal components can exploit this mechanism to evade immune surveillance [5]. Evidence from in vivo studies supports this immunomodulatory role. Rubinstein et al. (2004) and Nambiar et al. (2019), using murine tumour models, independently demonstrated that Gal1 facilitates tumour immune evasion by selectively modulating T cell responses [9,23]. Specifically, Gal1 was shown to induce apoptosis and functional inactivation of CD4⁺ T cells, while sparing CD8⁺ T cells, thereby reshaping the immune landscape to favour tumour progression. Notably, the inhibition of Gal1 enhanced tumour rejection and stimulated robust, tumour-specific T cell immunity [9]. Furthermore, Gal1-deficient mice exhibited resistance to rechallenge with wild-type, Gal1-expressing tumours, highlighting Gal1’s central role in tumour immune evasion. These findings highlight Gal1 as a key contributor to the establishment of an immunosuppressive microenvironment and a promising immunotherapeutic target with the potential to restore immune surveillance and improve cancer treatment outcomes.

## 3. The Immunomodulatory Role of Gal1 in the TME

Gal1 has been shown to suppress the immune system in the TME, and although this has been investigated, very little is known about the molecular mechanisms. The TME is a complex structure comprised of a variety of cell types such as ECs, pericytes, fibroblasts, tumour cells, and immune cells [24]. Malignant cells affect their microenvironment through the release of extracellular signals, which stimulate angiogenesis, proliferation, and avoid detection from the immune system by immune tolerance mechanisms [24]. As such, tumour cells could evolve to evade the immune surveillance, aiding tumour progression and metastasis. Immunosuppressive molecules such as Gal1 are vital in triggering such immune cell function suppression [25]. This section will discuss how Gal1 is involved in immunomodulation in the TME to favour tumour progression.

### 3.1. Suppression of T Cell Function

Tumour-reactive T cells exert effector functions that enable them to mount immune responses against malignant cells. The phenotypic alterations in cancer cells, including the aberrant expression of tumour-associated antigens, facilitate their recognition by tumour-infiltrating lymphocytes (TILs). Upon activation, TILs mediate antitumour activity through multiple mechanisms, notably the secretion of pro-inflammatory cytokines that disrupt tumour growth and promote immune-mediated tumour clearance [26,27]. TILs are actively recruited to the tumour site, where their extravasation involves a multistep process beginning with interactions between T cells and activated ECs, followed by transendothelial migration (TEM). In prostate cancer, elevated expression of Gal1 by ECs has been implicated in the inhibition of T cell TEM. In vitro studies showed that human umbilical vein endothelial cells (HUVECs), when exposed to conditioned media derived from prostate cancer cells, exhibited significantly increased surface-bound and secreted Gal1. Under these conditions, a significant reduction in T cell transmigration across the endothelial monolayer was observed [28]. Importantly, this upregulation of Gal1 did not affect T cell adhesion to the endothelial surface, suggesting a specific inhibitory effect on the transmigration phase of T cell extravasation.

In the head and neck cancer (HNC) model, the adoptive transfer of T cells from Gal1-knockout (KO) tumour exhibited a significant increase in live CD3^+^ T cells in the harvested tumour compared to the adoptive transfer from Gal1-wild type (WT) tumour [23]. In addition, the mechanism by which Gal1 affected the T cells’ translocation was focused on its receptor, CD43 (He and Baum, 2006 [28]). CD43 is believed to play an important role in mediating cell migration due to its localisation at the uropod region of the migrating lymphocytes [29]. The clustering of CD43 on T cells upon the interaction with Gal1 may inhibit the localisation of the receptor at the uropod, thereby preventing T cells TEM [28]. However, the location and distribution of CD43 clusters in T cells remains unclear. As there are multiple Gal1 receptors on T cells, further research is needed to validate that the clustering of CD43 can solely impact T cell migration.

Another proposed mechanism by which Gal1 suppresses the T cell response against tumour cells is through the immune checkpoint proteins. Tumour-secreted Gal1 resulted in the upregulation of immune checkpoint molecules on ECs, namely, programmed death ligand 1 (PD-L1) [23]. As PD-L1 binds to programmed death protein 1 (PD-1) receptor on T cells, the PD-1/PD-L1 pathway can inhibit both innate and adaptive immune cell functions, triggering the tumour-mediated immune suppression in the TME [30]. Upregulated in the TME, the pathway has become one of the promising targets for the immune checkpoint inhibitors (ICIs) to nullify its suppression on the immune cells. Nevertheless, resistance to ICI treatment in cancer patients has been demonstrated. Nambiar et al. (2019) indicated that increased PD-L1 expression in murine ECs is observed when co-cultured with Gal1 WT tumour cells, while such an increase was not observed when ECs were co-cultured with Gal1 KO tumour [23]. It is possible that Gal1 induces PD-L1 expression through the Janus kinase/signal transducers and activators of transcription (JAK/STAT) pathway. Increased phosphorylated STAT1 and JAK2 were seen in the co-culture of mouse ECs and Gal1 WT-containing conditioned media [23]. PD-L1 can also be expressed on other cells in the TME, such as tumour-associated macrophages (TAMs). It has been demonstrated that Gal1 can bind to its receptor, leading to JAK/STAT pathway activation, thus upregulating PD-L1 expression on TAM cell surface [31,32]. On the other hand, treatment with anti-Gal1 antibody partially reversed the resistance to PD-L1 blockade by reducing the tumour volume and increasing the number of CD8^+^ T cells in the tumour [23]. Combinatorial treatment with anti-Gal1 and PD-L1 inhibitors could restore the inhibitors efficacy and suppress the therapeutic resistance due to Gal1 presence in the tumours. The approach on Gal1 in tackling the ICI treatment resistance is promising and thus warrants further evaluation across various types of cancers.

Apart from Head and Neck Squamous Cell Carcinoma (HNSCC), Gal1 immunomodulatory function, particularly involving T cell apoptosis, was also observed in other types of cancer, such as skin, pancreatic, and brain cancers. The apoptosis rate was shown to be higher in the activated splenocytes incubated with supernatant from melanoma cells with high Gal1 expression [9]. Co-culture of activated T cells with pancreatic stellate cells (PSCs)-derived Gal1 resulted in an increase in caspase-3 and caspase-9 activity [33]. Moreover, the increased activation of caspase-3 in T cells can be seen when co-cultured with Gal1-positive glioma cells or Gal1-transfected HeLa cells [34]. Both caspase-3 and -9 are involved in activation-induced cell death (AICD), which triggers T cell apoptosis following their activation and pathogen clearance [35]. Since cell–cell interaction is required to effectively stimulate T cell apoptosis, Gal1 expressed on the tumour cell surface needs to come into direct contact with the T cells [34]. Nevertheless, the molecular mechanism by which Gal1 binds to T cells leads to the activation of the caspase-mediated apoptosis pathway remains to be clearly understood. Interestingly, Gal1 is highly expressed in the stromal cells of the poorly differentiated pancreatic cancer tissue, while the stromal cells in a well-differentiated pancreatic cancer have shown lower expression of Gal1, suggesting a key role for Gal1 in metastasis [33].

Gal1 has also been shown to sensitise resting T lymphocytes to Fas/FasL-mediated apoptosis, probably through activation of caspase-8 [36,37]. Gal1 triggers apoptosis by activating the caspase cascade, which has the potential to initiate alterations in cell form and structure and cause mitochondrial membrane depolarisation [36,38]. These effects are linked to the production of ceramide when p56lck and ZAP70 are present [36].

Gal1 has also been shown to induce mitochondrial budding and fission, evidenced by a significant upregulation of fission-associated proteins such as h-Fis and DRP-1. In parallel, Gal1 was found to promote mitochondrial coalescence, suggesting a complex role in mitochondrial dynamics. These effects were observed in both resting and activated human T lymphocytes, further supporting the immunomodulatory function of Gal1 in shaping T cell behaviour through metabolic and structural reprogramming [36]. This response is dose-dependent as the cumulative concentration of Gal1 significantly sensitised human T lymphocytes to anti-Fas-mediated apoptosis [36]. Despite normal T cells generally being resistant to apoptosis, an increased concentration of Gal1 was a powerful inducer of apoptosis in human resting T lymphocytes. These findings indicate that Gal1 enhances Fas/CD95-mediated apoptosis, leading to the cell death of human resting T lymphocytes [36]. Although caspase-8 has been shown to be involved, Hahn et al. (2004) reported that apoptosis induced by Gal1 was mediated by a caspase-independent pathway, through the translocation of endonuclease G from mitochondria to the nucleus [39]. Thus, Gal1-induced T cell apoptosis requires further investigation for therapeutic development.

The family of galectins has been found to modulate an extensive range of cytokines in immune cells [40]. Different sets of cytokines can induce the differentiation of naïve T cells into various types of helper T cells, including T helper 1 (Th1) and T helper 2 (Th2) cells. When activated, Th1 cells are responsible for the pro-inflammatory cytokine production, which contributes to the priming of cytotoxic CD8^+^ T cells and the anti-tumour immune response. On the other hand, activated Th2 cells produce immunoregulatory cytokines such as interleukin (IL) 4 and IL-10, which regulate the responses mediated by Th1 cells and cytotoxic T cells. Thus, a shift in Th1/Th2 balance in cancer is responsible for the immune suppression and progression of tumours [41,42]. Studies have reported substantial effects Gal1 has on Th1 and Th2 cells. For instance, Gal1 expression in patients with leukemic cutaneous T cell lymphoma (L-CTCL) has been found to promote Th2 skewing of non-malignant T cells, as IL-4 was markedly increased (*p* < 0.001 [43]). Meanwhile, the level of IFNγ, produced by Th1 cells, significantly decreased in both L-CTCL and in vitro mouse models when co-incubated with Gal1 [43,44].

In pancreatic cancer, an increase in IL-4 and a decrease in IFN-γ cytokine production can be observed when T cells are co-cultured with Gal1-overexpressing PSCs [45]. An increase in IL-4 production has been shown to play a role in cancer cell growth and progression. Another study investigating prostate cancer showed that expression of IL-4 increases the clonogenicity of benign primary prostate cells. The subunit of its heterodimeric receptor complex, IL4Rα, was also shown to be heightened in its expression in the malignant tissue [46]. IFN-γ, however, contributes to the antitumor immunity through its role in Th1 polarisation, induction of cancer cell apoptosis, inhibition of angiogenesis, and modulation of tumour senescence [47]. By skewing T cell polarization towards a Th2 phenotype and suppressing Th1 differentiation, Gal1 emerges as a key immunoregulatory factor and a major driver of cancer cells’ escape from immune surveillance. 

Gal1 has been reported to suppress CD8^+^ T cell function. The study conducted by Gandhi et al. (2007) demonstrated that Gal1 induced a reduction in infiltration, proliferation, and IFN-γ expression in Epstein–Barr virus-specific CD8^+^ T cells in Hodgkin lymphoma [48]. Moreover, exosomes released by various HNC cells were found to express Gal1, which induced a suppressive phenotype in CD8^+^ T cells, characterized by the downregulation of CD27/CD28 expression [49]. Multiple studies have also corroborated that tumour-derived Gal1 undermines the anti-tumour efficacy of cytotoxic T cells [9,22,50]. Intriguingly, endogenous Gal1, primarily synthesized by CD8^+^ T cells, was shown to impair their own cytotoxic functionality and proliferative capacity in a murine model of prostate cancer [51]. Hence, Gal1 appears to function as a negative autocrine regulator of activated CD8+ T cells, potentially involving the antagonistic modulation of extracellular signal-regulated kinase (ERK) signalling activated by the T cell receptor (TCR) [52]. In summary, the cumulative evidence suggests that various sources of Gal1 can effectively impede cytotoxic T cells, which serve as pivotal tumour-killing effectors, thereby establishing an immune-privileged environment within tumours.

Gal1 has been shown to regulate immune responses by modulating the function of dendritic cells (DCs), impairing their maturation, and enhancing their tolerogenic properties [53,54]. The tolerogenic effects of Gal1 on DCs are mediated, in part, by promoting the production of the immunosuppressive cytokine interleukin-27 (IL-27), which subsequently inhibits the production of interferon-gamma (IFN-γ) and IL-17, leading to a weakened T cell response. IL-27 also acts directly on T cells to induce the production of IL-10, thereby suppressing T cell activation. Furthermore, Gal1-induced IL-27 can facilitate the differentiation of type 1 regulatory T cells (Tr1), which are known to suppress inflammation and contribute to immune evasion and tumour progression in mouse models [53,54].

In the context of lung cancer, Gal1 has been found to induce IL-10 production in monocytes and monocyte-derived DCs. This not only diminishes T cell activation but also increases the immunosuppressive CD4⁺CD25⁺FOXP3⁺ Treg population [55]. Upregulation of IL-10 was also observed in tumour-infiltrating DCs of human lung cancer samples and in mice transplanted with lung cancer cells. A significant reduction in IL-10-producing, tumour-infiltrating DCs and lung cancer incidence was evident in mice harbouring Gal1-silenced tumour cells, emphasizing that Gal1 is a potential target for the development of lung cancer therapeutics [55].

### 3.2. Gal1-Induced Suppression by Regulatory T Cells

**Figure 1:**
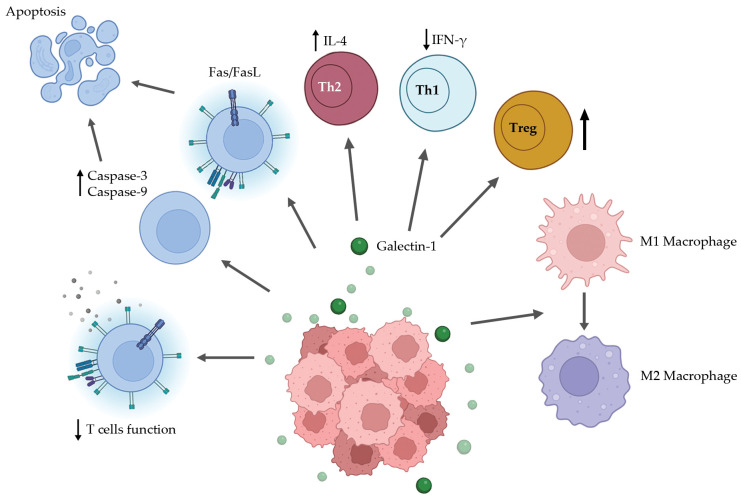
Immunomodulatory action of Gal1 within the TME. Gal1 is secreted by the tumour cells as well as the surrounding tissue and interacts with several immune cell types to downregulate the host immune response against the malignant cells. Firstly, Gal1 excludes T cells from the microenvironment and promotes their apoptosis via the upregulation of caspase signalling as well as sensitising T cells to Fas/FasL signalling. Gal1 also interacts with T helper cells, inducing decreased production of IFN-γ by Th1 and increased levels of IL-4 in Th2. Lastly, Gal1 upregulates Treg activity and promotes the phenotypic change in macrophage from M1 to M2 phenotype.

Another essential component that contributes to the immunosuppressive tumour environment is the presence of regulatory T cells (Tregs) in the TME (Figure 1). The recruitment and induction of Tregs in the TME lead to both contact-dependent and independent suppression of anti-tumour response [56]. An increased percentage of CD8^+^ Tregs can be observed in the spleen, draining lymph node (DLN), and tumours of the wild-type syngeneic colorectal cancer (CRC) mouse model. In contrast, there were significantly lower numbers of CD8^+^ Tregs from the spleen, DLN, and tumours of the Gal1 knockdown CRC mouse model (*p* < 0.05 [57]). Moreover, a reduction in IL-10 production can be seen when CD8^+^ Tregs, isolated from the Gal1 knockdown CRC mouse model, were co-cultured with splenocytes from the healthy WT mice compared to the CD8^+^ Tregs isolated from WT CRC mice [57]. Activated CD4^+^ CD25^+^ Tregs were shown to express high levels of Gal1. In addition, the inhibition of Gal1 using anti-Gal1 antibody reversed the in vitro suppressive activity exerted by Tregs [58]. This highlights the importance of Gal1 in the inhibitory functions of both CD4+ and CD8+ Tregs. Further research on how Gal1 influences Treg functions in the cancer setting could expand our understanding of Gal1’s role in immune escape mechanisms and cancer progression. Targeting Gal1 in the TME could potentially suppress Treg functions, allowing the activation of anti-tumour immunity. 

Gal1 has been shown to influence various aspects of T cell functionality. The majority of its roles serve to bolster cancer progression, as illustrated in HNSCC, pancreatic cancer, leukaemia, and many more. This section explored how Gal1 expression enhances T cell-related factors that contribute to cancer progression. An in-depth exploration of the cellular mechanisms underlying Gal1 functions could be further elucidated. Ultimately, investigating how Gal1 interacts with its receptor to modulate signalling pathways in T cells proves to be an intriguing question to understand its contribution towards the suppressive TME. 

### 3.3. Gal1 Induced Modulation of Macrophages

Contrary to blocking T cells, Gal1 was reported to increase the M2 phenotype of macrophages (Figure 1). Abebayehu et al. (2017) used polydioxanone (PDO) fibre scaffolds, which promoted an M2 phenotype in cultured macrophages, and studied the effect of Gal1 on an M2 macrophage response [59]. The study found that Gal1 increased arginase-1, a manganese-containing enzyme, whilst reducing iNOS and IL-6 production in macrophages derived from the bone marrow of mice compared to PDO alone [59]. Gal1 exhibited comparable potency to IL-13, a well-established M2 macrophage polarisation inducer, in regulating arginase-1 expression and modulating cytokine production in macrophages [59]. When preventing ERK mitogen-activated protein kinase, this had no effect on the impact of Gal1 on arginase-1 and iNOS; however, it did reverse the suppression of IL-6, indicating that IL-6 is regulated by a different mechanism [59]. Additionally, tumour cell-secreted Gal1 was also reported to induce M2 phenotype in TME during the early stages, with the activation of immune checkpoints including PD-L1/CD274 and IDO1 [32]. Such induction of the M2 phenotype indicates a pro-tumorigenic role of Gal1 in the TME.

Gal1 is also highly expressed in the stroma of surrounding tumour cells, such as TAMs. Davuluri et al. (2021) reported that TAMs are involved in the active secretion of Gal1 in response to stimuli from hepatocellular carcinoma (HCC) cells [60]. Secreted Gal1 led to an increase in the systemic level of Gal1 and the tumour growth of HCC in mice [60]. This secretion was shown to be mediated by TLR2-dependent secretory autophagy, with Gal1 operating as cargo of autophagosomes interacting with multivesicular bodies via Rab11 and VAMP1 [60]. Autophagy-regulated Gal1 release in TAMs correlated with reduced survival of patients with HCC [60]. Accumulation of Gal1 in the HCC stroma has been found to be positively associated with the size of the tumour, stage of cancer, and level of metastasis [60]. In response to stimuli from cancer cells or TME, stromal cells release Gal1, which exerts paracrine effects that support the development of cancer [60]. For example, Chong et al. (2016) showed that Gal1 produced from fibroblasts stimulated the migration and invasion of both breast and gastric cancer cells by increasing the expression of matrix metallopeptidases or promoting epithelial-mesenchymal transition (EMT) [61]. These findings underscore the pivotal role of Gal1 in modulating macrophage function and broader immune signalling pathways.

### 3.4. Gal1 Inhibition Can Enhance the Efficacy of Existing Immunotherapies and Help Overcome Treatment Resistance

Wang et al. (2024) showed that prostate cancer cells secrete Gal1, which promotes immune evasion by inducing T cell apoptosis [62]. They developed LLS30, a benzimidazole-based small molecule that binds to the CR domain of Gal1, thereby inhibiting its interaction with the CD45 receptor and suppressing Gal1-mediated T cell apoptosis. This inhibition not only promotes T cell infiltration into the tumour but also enhances the efficacy of anti-PD-1 immunotherapy. Combined treatment with LLS30 and anti-PD-1 led to increased CD8⁺ T cell infiltration and improved tumour regression.

Liu et al. demonstrated that Gal1 targeted PET can be used to predict immune checkpoint blockade (ICB) efficacy before therapy. They developed a Gal1 specific radiotracer 124I-αGal1, which showed high stability in vitro and Gal1 binding specificity. This has been done by treating tumour-bearing mice with albumin-bound paclitaxel, which can upregulate Gal1 expression. Results have showed a higher uptake of 124I-αGal1 in the tumours treated with albumin nanoparticle than in control at 24 and 72 h [63].

Liu et al. also designed a Gal1 inhibitor-loaded hydrogel scaffolds that could act inside the immunosuppressive tumour microenvironment. Their results highlighted that combining a Gal1 inhibitor such as TDG (thiodigalactoside) with anti-PD-1 and anti-CTLA-4, both ICB treatment, was more effective than using ICB on its own [63].

## 4. Gal1 as a Potential Therapeutic Target

In addition to their pro-tumorigenic and immunomodulatory roles discussed above and elsewhere [64,65], Gal1 has emerged to be a key mechanism for chemoresistance in various cancers [66]. For instance, Gal1 was shown to confer resistance to doxorubicin in HCC through mechanisms involving the modulation of P-glycoprotein [66]. This highlights the importance of targeting Gal1 to reduce tumour growth as demonstrated by Gal1 knockdown in breast cancer cells, which significantly reduced tumour burden with a marked decrease in tumour growth [11]. Furthermore, Gal1 silencing led to the inhibition of lung metastasis by inhibition of Treg cell expansion and/or down-modulation of their suppressive activity, with fewer colonies observed compared to the controls [11]. Thereby, it is now imperative to develop therapeutic interventions against Gal1. Several in vitro and pre-clinical studies have shown promise with some advancements for potential clinical trials, as discussed below (Table 2).

### 4.1. Non-Carbohydrate Gal1 Inhibitors

The development of carbohydrate-based Gal1 inhibitors has encountered several limitations, including high aqueous solubility, suboptimal pharmacokinetic profiles, and limited target selectivity [90]. To overcome these challenges, research has pivoted towards the design of non-carbohydrate Gal1 inhibitors, which have demonstrated high binding affinity and favourable pharmacodynamic characteristics, as supported by data from NMR spectroscopy, HPLC, fluorescence assays, and computational simulations [90].

Sethi et al. (2021) synthesised a series of 22 morpholine-linked coumarin–triazole hybrid compounds as potential non-carbohydrate inhibitors of Gal1 [91]. In this structural framework, the morpholine moiety functioned as a sugar mimic, while the coumarin and triazole rings substituted for the carbohydrate core and imparted cytotoxic properties. These compounds were evaluated for cytotoxicity against various cancer cell lines derived from bone, lung, breast, colon, and liver tissues, alongside normal embryonic fibroblast controls. Among them, compounds 14 and 15 emerged as lead candidates based on their ability to engage critical residues within the CRD of Gal1, such as Trp68, and form extensive hydrogen-bond networks. However, in silico predictions flagged potential hepatotoxic effects [91].

Compound 14 was particularly efficacious, inducing cell cycle arrest in the sub-G1 phase, promoting apoptosis in osteosarcoma cells, and eliciting a marked increase in reactive oxygen species alongside a reduction in mitochondrial membrane potential. Enzyme immunoassays confirmed the suppression of Gal1 overexpression following treatment. Structurally, compound 14 established robust hydrogen-bond interactions through its coumarin oxygen, morpholine, and triazole moieties with residues Ser29, Asn33, and Arg48, in addition to forming stable interactions with His44, His52, and Trp68, thereby anchoring the molecule within the Gal1 active site [90,91].

Similarly, compound 15 demonstrated strong binding affinity within the Gal1 CRD, mediated by hydrogen bonding between its coumarin oxygen and thiazole components with residues such as Asn61 and Gly69, and reinforced by additional interactions with His52 and Trp68 [91]. These findings demonstrate the therapeutic potential of morpholine-linked coumarin–triazole and coumarin–thiazole hybrid compounds as selective Gal1-targeted anticancer agents.

### 4.2. OTX008—A Selective Gal1 Inhibitor

OTX008 is a derivative of calixarene that binds the Gal1 amphipathic beta sheet formation, and therefore can act as a selective small-molecule inhibitor of Gal1. It has been shown to downregulate cancer cell proliferation, invasion, and angiogenesis [92,93]. This molecule displays its anti-tumour activity by inhibiting the cell cycle, as well as enhancing the cytotoxicity of chemotherapy [87]. Dings et al. (2006) reported the inhibitory role of OTX008 in vitro in inhibiting both proliferation and migration of ECs and in the developing tumours in melanoma and ovarian cancer models [94]. For instance, the addition of OTX008 induced a significant reduction in micro-vessel density, demonstrating an anti-angiogenic effect in endothelial cell-induced tube formation [94]. Contrary to this, an increase in the frequency of pericytes was observed in OTX008-treated mice [94]. This allowed for more blood vessel remodelling and the ratio between ECs and pericytes to be corrected, as cancer patients normally show an increase in ECs compared to pericytes [94,95,96]. Thus, with an influx of pericytes, the blood vessels became less leaky and reduced inflammation. Similar to these results, Astorgues-Xerri et al. (2014) studied the effects of OTX008 on human cancer cell lines in vitro and found that it inhibited the proliferation of cultured tumour cells [87]. The results correlated with the expression of Gal1 across the cell lines, with those expressing epithelial differentiation markers showing greater sensitivity to OTX008 compared to mesenchymal cells [87]. In addition, OTX008 was reported to hinder tumour invasion by suppressing the expression of Gal1 and inhibiting ERK1/2 and AKT-dependent signalling pathways. It also induced G2/M cell cycle arrest through CDK1 inhibition [87]. In vivo, xenograft models were analysed, and results showed OTX008 to downregulate Gal1 in the treated tumour and reduce micro-vessel density and tumour burden [87,97]. However, further clinical investigation is required to assess its therapeutic benefits in humans.

### 4.3. Anti-Gal1 Monoclonal Antibodies

Due to the potent immunosuppressive and pro-angiogenic effects of Gal1, further research has been carried out in targeting Gal1 therapeutically. For instance, recently, newly designed anti-Gal1 monoclonal antibodies (anti-Gal1 mAbs) were investigated in vitro to block Gal1-mediated cellular functions [98]. Interestingly, one of the antibodies significantly improved the immune responses in mice by blocking and inhibiting Gal1 [98]. It was also found that this anti-Gal1 mAbs significantly reduced EC migration and tube formation [98]. Furthermore, the study analysed the levels of CD4^+^ and CD8^+^ T cells and found that the addition of anti-gal1 mAbs increased the percentage of T cells [98], suggesting an increase in infiltration of T cells. In contrast, Pfeffer et al. tested a newly designed mAb against Gal1 and found that, although it bound to cell surface-bound Gal1, it did not block Gal1 activity in mediating Treg cell contact-dependent suppression [99]. Therefore, further investigation is required to evaluate the inhibitory activity of anti-Gal1 mAbs. Nevertheless, monoclonal antibodies have been in clinics as targeted cancer therapy (e.g., Avastin (bevacizumab, anti-angiogenic)) and Herceptin (trastuzumab, anti-HER2 receptor in HER2-positive breast cancers) [100], and, therefore, anti-Gal1 mAbs research should be encouraged with an aim for inclusion in future clinical studies.

### 4.4. Vaccinations Against Gal1

Considering the efficacy of anti-Gal1 monoclonal antibodies, vaccinations that stimulate the immune system to produce polyclonal anti-Gal1 antibodies represent an additional therapeutic strategy. The administration of TRX-mGal1, recombinant Gal1 protein, led to an increase in anti-Gal1 antibody as well as an improvement in tumour vascular perfusion [72]. Gal1-immunised mice also exhibited an increase in M1 macrophages and CD8+ T cells, of which their cytotoxic activity was also heightened (*p* < 0.05 [72]). As one of the key immunoregulatory roles of Gal1 is to impair the immune cell recruitment to TME, the Gal1 vaccine has been shown to improve leukocyte recruitment in vivo. Specifically, an increase in CD8^+^ T cells in the tumour indicates that the administration of the vaccine has hindered Gal1’s ability to inhibit the migration and functional activity of T cells. Authors have shown that the efficacy of TRX-Gal1 to restore various immune cell activities agrees with the observed effects when using the monoclonal anti-Gal1 antibody [71,72,80]. Nevertheless, safety, specificity, and bioavailability of the vaccine remain crucial factors that cannot be neglected. Gal1 neutralisation in the mouse model exhibited a preeclampsia-like syndrome; therefore, the vaccine may not be suitable for use in pregnant women [101]. The overall reduction of circulating Gal1 following the vaccination could also lead to undesirable side effects, while the specificity of the antibody to Gal1 in the TME has not been well-understood. Although TRX-Gal1 has shown promising results in inhibiting key Gal1 functions and reducing the tumour burden, the vaccine still requires further elucidation on its safety, selectivity, and efficacy [72].

## 5. Future Perspective

Gal1 might potentiate current cancer treatments and increase their therapeutic efficiency. As an emerging target, Gal1 has the potential to act as a drug target in the treatment of cancer; however, with gaps in knowledge, more research is necessary to provide safer and more selective and specific treatment options. Most studies analysing the inhibition of Gal1 as a therapeutic agent in cancer are still in pre-clinical trials; thus, information is absent on patients’ safety. Pre-clinical research should focus on the pharmacokinetics, toxicity, and possible side-effects of prescribing drugs targeting Gal1 to human patients. In the context of future cancer therapies, OTX008, along with anti-Gal1 monoclonal antibodies and vaccines, demonstrates significant potential as a novel class of therapeutic agents. 

With a critical need for biomarkers in cancer for early detection and intervention, the emergence of Gal1 as a possible biomarker could hold potential in cancer prognosis. Masoodi et al. analysed Gal1 as a biomarker in ovarian cancer and observed levels of Gal1 to be elevated in epithelial ovarian tumours; contrary to a decrease in Gal1 in post-chemotherapy and surgery [13]. Additionally, Nanami et al. (2021) studied the prevalence of serum Gal1 autoantibodies in seven different types of cancer and found that patients with hepatocellular carcinoma and lung cancer display significantly higher positive levels of serum Gal1 antibodies [102]. Furthermore, Gal1 was highly expressed in malignant thyroid lesions in mice, compared to benign lesions [17]. This displays Gal1 as a reliable diagnostic marker for thyroid cancer. Likewise, in a recent study using multivariate Cox regression analysis in ovarian and endometrial cancer patients, we found that the expression of Gal1 displayed a significant prognostic value [7,103]. These facts demonstrate that therapeutic targeting of Gal1 expression and release in the TME could potentially improve overall survival. Furthermore, the observation of Gal1 as a potential biomarker within the TME and plasma demands further clinical research.

## 6. Conclusions

With both the immunomodulatory and proangiogenic roles of Gal1 within the TME made clear with extensive research, the importance of Gal1 has been considerably highlighted. Ongoing research into the therapeutic strategies in targeting Gal1 presents numerous possibilities for future treatments of cancer using Gal1 as a target. There is therapeutic potential in targeting interactions between Gal1 and glycan to overcome immunosuppression in cancer and strengthen antitumoural immunity, as well as treatments aimed to stimulate immunoregulatory processes driven by Gal1 to lessen inflammation. The results from numerous studies analysing the immunosuppressive and pro-angiogenic influence of Gal1 on tumour growth, proliferation, and apoptosis have evidently demonstrated the stark possibilities that Gal1 presents as a cancer therapeutic target. With future research already in practise, the evidential promise this shows is extremely positive for cancer therapies and underpins an exciting era of possibility for novel cancer therapeutic approaches.

## Figures and Tables

**Table 1:** A table displaying the cancer types in which Gal1 is upregulated, the associated phenotypes (Gal1 Upregulation Effects) observed in each cancer type, and the outcomes of strategies targeting Gal1 and its downstream effectors for potential therapeutic approaches and as a biomarker for early detection.

Cancer Type	Gal1 Upregulation Effects	Gal1 Targeting Outcomes	Refs
Melanoma	↑ Tumour growth ↓ Th-1 anti-tumour response	- Gal1 depletion improves the melanoma cells’ susceptibility to autophagy induced by temozolomide, a chemo-therapy drug.	[9,10]
Breast	↑ Aggression of human breast tumour ↑ Tumour growth and metastasis ↑ Frequency of Treg cells including: CD4+, CD25+ and Foxp3+	- Gal1 knockdown leads to decreased cell proliferation, migration, and invasion in vitro in breast cancer model	[11,12]
Epithelial Ovarian Cancer (high-grade serous carcinoma)	↑ Cell proliferation ↑ Angiogenesis	- Gal1 as a biomarker for early detection - Inhibiting Gal1-activated ERK1/2 and PI3K pathways reduced proliferation and migration of human omental microvascular ECs	[7,13]
Non–small cell lung cancer	↑ COX-2 expression and prostaglandin E2 to stimulate tumour progression ↑ Chemotherapy resistance ↑ Cell migration and invasion	- Gal1 as an innovative target for combined modality therapy in lung cancer	[14]
Castration-Resistant Prostate Cancer	↑ Cell adhesion ↑ Cell migration ↑ Tumour invasion	- Gal1 inhibitors, e.g., LLS30 reduced tumour metastasis in vivo and improve the anti-tumour effect of chemotherapy drug.	[15]
Urinary bladder urothelial carcinoma cell	↑ Cell proliferation ↑ Tumour invasive capability ↑ Clonogenicity	- Silencing Gal1 and targeting the Gal1 mediated MAPK signalling pathway could present a novel therapeutic strategy for bladder cancer treatment	[16]
Follicular and papillary carcinomas	↑ Cell proliferation ↑ Cell migration ↑ Invasion ↑ Tumour growth	- Gal1 as a potential immunohistochemical marker to monitor tumour progression and distinguish benign thyroid nodules and malignant tumours.	[17]
Pancreatic ductal adenocarcinoma	Induction of apoptosis of CD4+ and CD8+ T cells ↑ Th2 cytokine secretion from T cells ↑ Tumour growth	- Gal1 knockdown increased CD4+ and CD8+ T cell numbers - Targeting Gal1 could aid in the development of targeted immune therapies to enhance anti-tumour immunity - Gal1 as a potential biomarker	[18,19]
Cutaneous head and neck cancer with perineural spread	↑ T and B lymphocytes ↑ Fox-P3 expressing T cells Mediates tumour-host immune system interactions Creates an immune-privileged environment for tumour cells to evade host immunity	- Inhibiting the expression or function of Gal1 potentially restore immune function	[20]
Glioblastoma	↑ Brain-infiltrating macrophages ↑ Myeloid-derived suppressor cells	- Gal1 as a potential target to deplete myeloid cells from the TME - Inhibiting Gal1 expression may overcome tumour immune resistance and enhance immunotherapeutic strategies	[21]
Neuroblastoma	Induced T cell apoptosis Inhibition of DC maturation	- Combining Gal1 blockade with DNA vaccines targeting neuroblastoma antigens could improve the effectiveness of immunotherapy - Targeting Gal1 could have a dual benefit by inhibiting immune suppression and promoting anti-tumour immune responses	[22]

COX-2, cyclooxygenase-2; DC, dendritic cell.

**Table 2:** Current known therapeutics targeting Gal1 in different cancer types.

Cancer Type	Agents	Theraputic Strategy/Modality	Development Stage	Materials	References
Melanoma	Thiodigalactoside	A small molecuale that inhibits Gal1 activity via binding to its CRD	Preclinical	Disaccharides	[67,68,69]
	4-F-GlcNAc	A metabolic inhibitor that disrupts the biosynthesis of LacNAc (N-acetyllactosamine), thereby reducing the availability of Gal1 binding sites on cell surface glycoproteins	Preclinical	Glycan	[70]
	TRX–mGal1	A recombinant vaccine protein was developed by fusing bacterial thioredoxin (Trx) with mouse Gal1 (mGal1)	Preclinical	Murine Gal1 vaccine	[71,72]
	GM-CT-01 (Davanat)	It binds to Gal1, disrupting its interactions with glycosylated receptors on cell surfaces and hence modulating immune response	Clinical	Galactomannan	[73,74]
Ovarian	Anginex	Direct binding of the angiostatic agent to Gal1 alters its affinity for glycoproteins. Combatorial treatment with irofulven further reduce tumour growth.	Preclinical	β-peptide	[75,76,77,78]
Non-small cell lung cancer	AP-74 M-545	Designed to Specifically binds to Gal1, disrupting its interaction to glycosylated receptors	Preclinical	Single-stranded DNA aptamer	[70]
Prostate carcinoma	LLS30	An inhibitor that binds to CRD, preventing Gal1 binding to CD45 and leading to the suppression of T cells apoptosis.	Preclinical	Small molecule	[15,62]
Kaposi sarcoma, prostate	8F4F8G7	Binding of the antibody to Gal1 inhibits Gal1-induced apoptosis of the activated T cells and cytotoxic T cells (CTL).	Preclinical	Monoclonal antibody	[79,80]
Head-neck cancer	GR-MD-02 (Belapectin)	Inhibits extracellular Gal1 and Gal3 from interacting with the glycosylated receptor on tumour cells.	Phase I clinical trial	Polysaccharide	[5,81]
Glioblastoma	Intranasal siRNA	The nanoparticles is capable of crossing the blood-brain barrier (BBB). Silencing Gal1 results in decreased tumour cell motility.	Preclinical	siRNA-loaded chitosan nanoparticles	[73,82,83]
Neuroblastoma	Minigene DNA vaccine	The Gal1 DNA vaccine can trigger the antitumour cytotoxicity.	Preclinical	DNA plasmid	[84]
Advanced solid tumours	OTX008	A small molecule that selectively binds Gal1 and inhibits cell cycle prgression and enhances tumour cell cytotoxicity	Phase I clinical trial	Calixarene compound	[85,86,87,88]
Metastatic colorectal cancer	GM-CT-01 (Davanat)	Treatment with GM-CT-01 observed an increase in IFN-γ secretion by T cells.	Phase II clinical trials	Polysaccharides	[74,89]
Breast cancer	Thiodigalactoside	A small molecuale that inhibits Gal1 activity via binding to its CRD	Preclinical	Disaccharides	[69]
Lymphoma	4-F-GlcNAc	A metabolic inhibitor that disrupts the biosynthesis of LacNAc (N-acetyllactosamine), thereby reducing the availability of Gal1 binding sites on cell surface glycoproteins	Preclinical	Glycan	[70]
